# Quantitative Trait Locus Mapping and Candidate Gene Identification for Fruit Acidity in Chinese Dwarf Cherry (*Cerasus humilis*) Using a High-Density Genetic Map

**DOI:** 10.3390/genes16101157

**Published:** 2025-09-29

**Authors:** Caizhen Guo, Fenglan Hu, Yuqi Li

**Affiliations:** Department of Biological and Food Engineering, Lyuliang University, Lishi 033000, China; 20081023@llu.edu.cn (F.H.); liyuqi7868@163.com (Y.L.)

**Keywords:** *Cerasus humilis*, genetic map, fruit acidity, quantitative trait locus mapping, candidate gene

## Abstract

Background/Objectives: The Chinese dwarf cherry (*Cerasus humilis*) is an endemic shrub fruit tree species in China. Its fruit is flavorful, nutrient-rich, and has considerable research and utilization potential. However, most currently cultivated varieties of *C. humilis* are highly acidic and primarily used for processing. Consumer-preferred, low-acid, fresh-eating varieties are scarce, limiting industrial development. We used 208 F_1_ individuals derived from a cross between high-acid “Nongda 4” and the low-acid “DS-1”. Methods: Restriction site-associated DNA sequencing (RAD-seq) was used to develop single-nucleotide polymorphism (SNP) markers and construct a high-density genetic linkage map. Using two years of fruit titratable acidity phenotypic data, quantitative trait locus (QTL) mapping and candidate gene screening were performed. Results: The genetic map contained 2491 SNP markers, assigned to eight linkage groups. The total genetic distance was 672.71 cm, with an average distance of 0.27 cm between markers, indicating high map quality. QTL mapping identified 18 loci associated with fruit titratable acidity, including 11 major-effect QTLs (logarithm of odds, LOD ≥ 3.5). These major-effect QTLs were concentrated on linkage groups LG2 and LG5, with an explained phenotypic variation of 8.6–31.13%. Two candidate genes were identified within QTL intervals: phosphoester phosphatase and MATE transmembrane transporter. The phosphatase gene’s expression showed a strong correlation with titratable acid content (*p* < 0.01, correlation coefficient 0.93), suggesting that it plays an important role regulating fruit acidity in *C. humilis*. Conclusions: This study supports marker-assisted breeding of low-acid, fresh-eating varieties, aiding commercial promotion of *C. humilis*.

## 1. Introduction

The Chinese dwarf cherry (*Cerasus humilis*) is a rare and endemic fruit tree resource in China. It has recently gained attention due to its unique biological characteristics and economic value [1,2,3]. The fruit of *C. humilis* is rich in vitamins, amino acids, carbohydrates, organic acids, and other substances [4]. *C. humilis* ranks among fruits with the highest calcium content, earning the title, “the star of calcium supplementation” [5]. It has high stress resistance (cold- and drought-resistant, barren-tolerant) and is valuable for ecological restoration (soil and water conservation) and fruit product development [6,7]. With the development of characteristic ecological agriculture, its cultivation area has gradually expanded. However, current cultivated varieties are highly acidic and primarily used for processing. Consumer-preferred, low-acid, fresh-eating varieties are scarce, impeding commercialization of *C. humilis*. Therefore, elucidating the genetic regulatory mechanism of fruit acidity and directionally improving fruit quality via molecular means are crucial for commercial promotion of *C. humilis* [8].

Genetic linkage maps are fundamental tools for indicating the genetic basis of fruit tree traits and also provide crucial support for quantitative trait locus (QTL) mapping [9,10]. The construction of maps relies on efficient molecular marker technologies [11]. Single-nucleotide polymorphisms (SNPs), due to their large quantity, wide coverage, and high detection efficiency, have become the mainstream choice for genetic map construction [12]. Many people have constructed genetic linkage maps using this method [13,14,15]. Restriction site-associated DNA sequencing (RAD-seq) is based on genome-wide restriction enzyme sites, with high-throughput sequencing conducted on specific restriction fragments [16]. This method considerably reduces genome complexity and sequencing costs, and rapidly identifies high-density SNP loci. It is widely used in genetic variation detection, genetic map construction, gene mapping for important traits, and population genetic evolution analysis [17,18]. This sequencing technique proves useful in examining the genetic mechanisms underlying target traits and has been widely applied to non-model organisms, with one or more genetic maps constructed in various fruit tree species [19].

Fruit acidity is a quantitative trait with complex genetic and variation mechanisms [20,21]. QTL mapping identifies chromosomal regions linked to target traits and estimates their genetic effects using association analysis of phenotypic data and marker genotypes. This provides a basis for marker-assisted selection, thereby improving breeding efficiency [22]. Diaz-Garcia et al. mapped 16 QTLs associated with titratable acidity in five cranberry fruit traits [23]. Tang et al. identified eight QTLs related to total acidity in jujube fruit using composite interval mapping, with LODs of 3.05–4.01 and a phenotypic variation explained (PVE) of 17.70–22.60% [24]. Jiang et al. used resequencing data to map 11 QTLs associated with fruit acidity in apricot and identified 88 genes related to malic acid, 310 related to citric acid, and 40 related to total acidity within QTL intervals [25].

Advances in molecular marker technologies have promoted the formulation of genetic linkage maps and have enabled effective QTL mapping [26,27]. Currently, it is widely used in various fruit trees, including pear [28], peach [29], plum [30], and apple [31]. However, studies on the genetic map and QTL mapping of *C. humilis* are limited, hindering progress in molecular marker-assisted breeding of *C. humilis*. *C. humilis* has fewer chromosomes (2n = 2x = 16) and a small genome (~229.21 Mbp) [32]. Constructing genetic maps for QTL mapping is essential for identifying genes related to fruit traits in *C. humilis*.

In the current study, 208 F_1_ hybrid individuals from the Nongda 4 × DS-1 cross were used as mapping materials. RAD-seq was used to develop SNP markers to construct a genetic map. QTL mapping for fruit titratable acidity (TA) and the screening of candidate genes were conducted. This study aimed to provide theoretical and technical support for molecular marker-assisted breeding and accelerate the development of breeding high-quality *C. humilis* varieties.

## 2. Materials and Methods

### 2.1. Plant Materials

All experimental materials were collected from the C. humilis Shixiang Experimental Field of Shanxi Agricultural University (37°26′ N, 112°32′ E). A F_1_ hybrid population of 208 individuals derived from a cross of Nongda 4 × DS-1 was used for this study. Nongda 4 is a high-acid cultivar selected from wild *C. humilis*. Its fruits are subglobose, with an average single-fruit weight of 6 g and a titratable acid content of 2%. This cultivar ripens in early September. DS-1 is a low-acid strain selected from wild *C. humilis*. Its fruits are subglobose, with an average single-fruit weight of 4 g and a titratable acid content ranging from 0.9% to 1%. This strain ripens in early August. Their hybrid progenies were planted in 2019. The experimental field had uniform water and fertilizer conditions, and field management was conducted referencing conventional field production management to ensure consistent growth environments for all plants.

### 2.2. RAD Sequencing and SNP Statistics

Young leaves of all samples were collected in spring and treated with liquid nitrogen. Genomic DNA extraction was performed with a DNA extraction kit (Solarbio, Beijing, China). Library construction and high-throughput sequencing were performed by Lianchuan Biological Company using the Illumina HiSeq PE150 platform (Illumina, San Diego, CA, USA). The quality-controlled sequencing data were aligned to the *C. humilis* reference genome (https://doi.org/10.6084/m9.figshare.11669673) using BWA software under default parameter settings, and the number of aligned reads was counted. Subsequently, variant calling was conducted using GATK and SAMtools [33]. SNPs consistently identified using both software were retained as reliable loci. Polymorphic SNPs were selected to form the final SNP dataset for subsequent genetic map construction.

### 2.3. Genetic Map Construction and Quality Evaluation

In this study, the female parent Nongda 4 had a fruit titratable acid content of 2.06–2.13%, while the male parent DS-1 exhibited a titratable acid content of 0.9%. Determination of fruit titratable acid content in F_1_ hybrid individuals showed that this trait followed a continuous normal distribution. These findings indicated that fruit acidity is a quantitative trait, which is suitable for subsequent QTL mapping [34].

The double pseudo-test-cross method was used for the construction of a genetic map [26]. To ensure genetic map quality, polymorphic SNPs were filtered and genotyped [35]. SNPs with a parental sequencing depth <5× were excluded. Selected markers whose genotypes covered at least 75% of all offspring individuals were retained, with thresholds adjusted appropriately based on marker data quantity. Markers with a chi-square test (*p* < 0.001) were filtered out to avoid impacting QTL mapping. As a cross-pollinated population was used, only polymorphic markers other than the aa × bb type (homozygous) were retained to match population characteristics.

SNP markers were divided into eight linkage groups based on their positions in the reference genome. JoinMap was used to determine the linear order of markers within linkage groups and calculate genetic distances between neighboring markers to produce the genetic map [36]. Genetic map quality was assessed by analyzing mapped marker linkage relationships. Visualization of these relationships was performed by generating plots using an in-house R script.

### 2.4. QTL Mapping and Screening of Candidate Genes

Using the constructed genetic map of *C. humilis* and phenotypic data collected over two consecutive years [34], QTL mapping for fruit titratable acidity in *C. humilis* was conducted using the interval mapping method in MAPQTL software (Version 7.0, Kyazma, Wageningen, The Netherlands) [25]. A threshold of LOD ≥ 3 was established to identify the presence of a QTL. QTLs with an LOD > 3.5 were considered significant. The proportion of PVE for each QTL was computed. QTL mapping intervals were matched against the physical positions of the *C. humilis* reference genome to obtain detailed information on genes within these regions. All genes within QTL intervals were functionally annotated using databases (NR, Swiss-Prot, COG, GO, KEGG) to screen candidate genes related to titratable acid metabolism in *C. humilis* fruit.

## 3. Results

### 3.1. RAD Sequencing Data Analysis

RAD-seq was conducted on two parents and 208 F_1_ individuals, generating 241.86 Gb of data. The maternal parent, Nongda 4, produced 0.79 Gb of data, with a base quality Q30 ratio of 91.15% and an average GC content of 41.61%. The paternal parent, DS-1, produced 1.78 Gb of data, with a base quality Q30 ratio of 90.87% and an average GC content of 41.7%. The 208 offspring produced 239.29 Gb of data, with an average base quality Q30 ratio of 90.65% and an average GC content of 40.82%. The average Q30 value of all samples was 90.89%, indicating that sample sequencing data were of good quality with high accuracy (i.e., low error rate) (Table S1). When aligned to the reference genome of *C. humilis* (Table S2), the maternal parent, Nongda 4, and paternal parent, DS-1, had an average sequencing depth of 5.89×, whereas that of the hybrid offspring was 5.25×; the alignment rates with the reference genome were 95.83%, 95.03%, and 93.71%, respectively.

### 3.2. Genetic Map Construction

**Figure 1:**
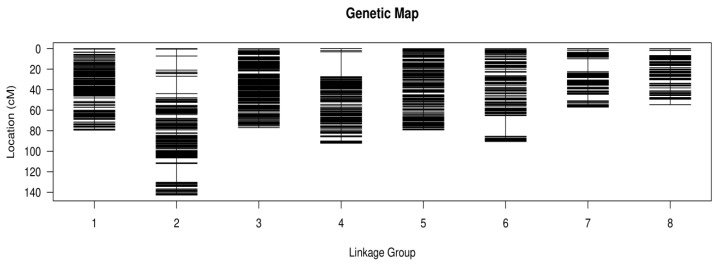
Genetic lengths and marker distribution of eight linkage groups in the genetic map of *C. humilis*. Black bars represent mapped markers.

After multiple rounds of rigorous filtering and genotyping, 2,491 SNP markers were finally confirmed to be usable. These included 1537 “lm × ll”, 668 “nn × np”, and 286 “hk × hk” marker types. A genetic map was constructed that included 2,491 SNP markers, with eight linkage groups (LGs) covering a total genetic distance of 672.71 cm (Table 1; Figure 1). The mean interval between markers was 0.27 cm. Linkage group lengths ranged from 54.6 cm (LG8) to 142.71 cm (LG2), with an average length of 84.09 cm per group. The number of SNP markers ranged from 153 (LG8) to 465 (LG1). LG2 was the longest linkage group, containing 142.71 SNP markers. The shortest was LG8, with 153 SNP markers. LG2 had the longest average genetic distance (0.37 cm), and LG1 had the shortest (0.17 cm). The average proportion of gaps < 5 cm in each linkage group was 99.49%.

### 3.3. Quality Evaluation of the Genetic Map

**Figure 2:**
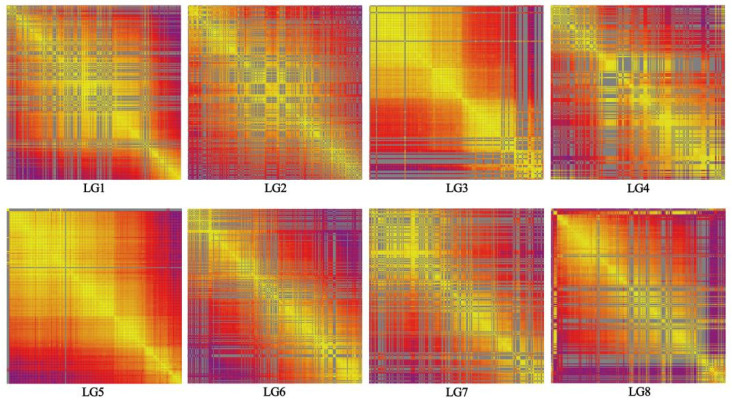
Marker linkage relationship on the genetic map. Each cell denotes the recombination rate of pair-wise markers. Yellow and purple indicate lower and higher recombination rates, respectively.

For assessing the quality of the genetic map, recombination plots were generated for each linkage group (Figure 2). A positive correlation was found between genetic distance and linkage strength across all linkage groups. This indicates that the constructed genetic map was of high quality.

### 3.4. QTL Mapping and Candidate Gene Identification

**Figure 3:**
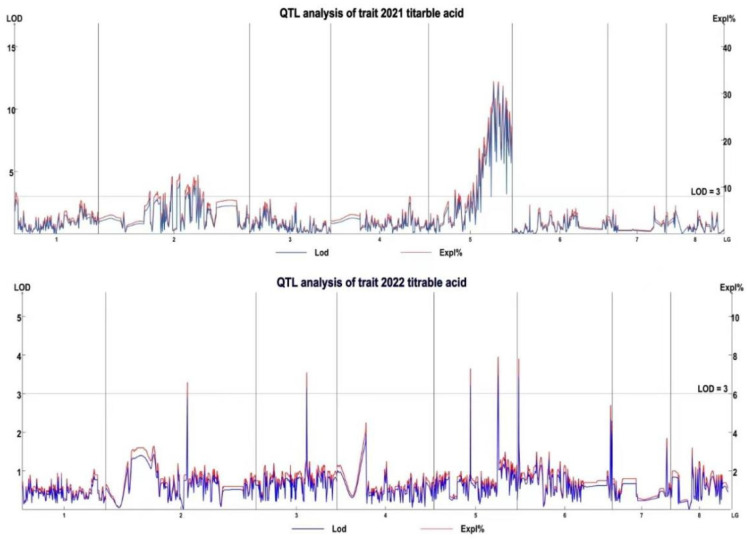
Quantitative trait locus (QTL) distribution for titratable acid on linkage groups. The *x*-axis represents the linkage groups.

QTL mapping results for titratable acidity in *C. humilis* fruit showed that 18 QTLs were detected in the 2021 or 2022 datasets (Table 2; Figure 3), with 11 being significant. In 2021, 13 QTLs were detected, which were mapped to linkage groups LG2 (21TA-1 to 21TA-7) and LG5 (21TA-8 to 21TA-13), with LOD thresholds of 3.25–11.28 and PVE of 10.19–31.13%. In 2022, five QTLs were identified, fewer than in 2021. These QTLs were each mapped to one of the linkage groups (LG1, LG2, LG3, LG5, and LG6), with LOD thresholds of 3.14–3.86 and PVE of 7.1–8.7%.

**Figure 4:**
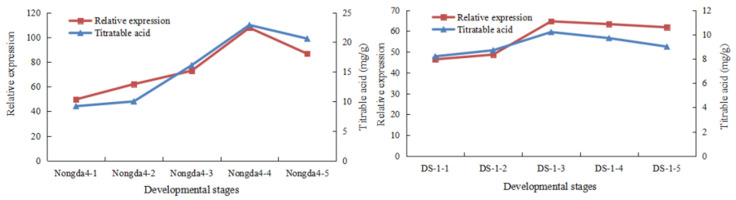
Dynamic changes of titratable acid content and phosphatidate phosphatase gene expression during the fruit development of different germplasms.

Based on the results of QTL mapping and database annotation, two candidate genes were identified. Both genes were located on LG2: a phosphoester phosphatase gene (*MSTRG.15867*) and a MATE transmembrane transporter (*MSTRG.17104*). Transcriptome data from Nongda 4 and DS-1 [8] were integrated to analyze the expression levels of candidate genes identified using QTL mapping in fruit of two *C. humilis* germplasms at different developmental stages. Simultaneously, a comparison was made with fruit titratable acid content at the corresponding stages (Figure 4).

As *C. humilis* fruits developed, the relative expression levels of the phosphoester phosphatase gene of both Nongda 4 and DS-1 increased and subsequently decreased, aligning with the changes in titratable acid content. Moreover, the expression level in Nongda 4 was higher than that in DS-1. According to the correlation analysis (Table 3), the relative expression level of the phosphoester phosphatase gene was strongly correlated with titratable acid content (*p* < 0.01), with a correlation coefficient of 0.93. This indicates that the gene is closely related to the regulation of fruit acidity in *C. humilis*.

## 4. Discussion

*C. humilis* not only has strong stress resistance but its fruit is rich in nutrients and holds considerable value in both ecological and economic aspects. However, high fruit acidity in existing cultivars limits their suitability for fresh consumption. Therefore, elucidating the genetic mechanism underlying fruit acidity and performing targeted improvements are essential for progress in commercial utilization. In this study, the experimental material consisted of an F_1_ hybrid population derived from crossing the high-acid Nongda 4 with the low-acid DS-1 varieties of *C. humilis*. A genetic linkage map was built utilizing RAD-seq technology. Two years of phenotypic data were used for QTL mapping of fruit titratable acidity and candidate gene identification. Genetic linkage maps are fundamental tools to indicate the genetic basis of fruit tree traits, and their density and accuracy directly affect QTL mapping efficiency [37].

In the current study, a genetic map was built using 208 F_1_ hybrids from a cross between Nongda 4 and DS-1, offering advantages in parental selection and population size. The titratable acid content of the maternal parent Nongda 4 is 2.06–2.13%, while that of the paternal parent DS-1 is 0.9%. There is a significant difference between the two parents (the difference is 1.16–1.23%). The titratable acid content of the 208 F_1_ populations varied greatly, ranging from 0.82 to 2.8, and showed a continuous normal distribution, which conforms to the characteristics of quantitative traits and lays a foundation for genetic map construction and QTL localization. The size of the F_1_ segregating population critically affects the map’s accuracy, resolution, saturation, and application. A larger segregating population is conducive to constructing a more accurate genetic map [29]. Large-scale cultivation of fruit tree populations requires a large amount of land area. Subsequent genetic linkage mapping also demands substantial investment in human resources, materials, and finances. This results in relatively small population sizes used for constructing fruit tree genetic maps. Currently, most constructed fruit tree genetic maps are based on populations of <150 individuals [23,24], which may reduce mapping precision. However, the 208 F_1_ individuals used in this study produced more molecular markers, substantially improving the map’s accuracy, resolution, saturation, overall representativeness, and reliability.

Traditional molecular marker technologies have single marker types and limited numbers, resulting in large gaps in constructed genetic maps, thus compromising map quality and subsequent research [38,39]. The advent of third-generation molecular SNP markers, genotyping by sequencing, and restriction enzyme digestion sequencing technologies has led to major advances in fruit tree genetic mapping [40]. RAD-seq, a reduced-representation genome sequencing approach based on genome-wide restriction enzyme digestion sites, reduces genome complexity and sequencing costs while enabling rapid identification of high-density SNP loci [41].

In the current study, RAD-seq technology generated 241.86 Gb of data, with an average Q30 value for all samples reaching 90.89%, indicating high sequencing quality data and low error rates, ensuring accurate SNP marker development. The constructed *C. humilis* genetic linkage map contained 2491 SNP markers. The eight linkage groups had a total genetic distance of 672.71 cm, with an average inter-marker distance of only 0.27 cm. Moreover, the average proportion of gaps < 5 cm per linkage group was 99.49%. This map provides a solid platform for subsequent QTL mapping and gene discovery. The mapping results align with successful RAD-seq application in other fruit trees, including apple [40], pear [41], and hawthorn [42].

QTL mapping is a key method for elucidating the genetic mechanisms of quantitative traits [43]; results are affected by multiple factors, with LOD score thresholds being particularly important [44]. The current study set an LOD score of 3.0 to detect QTL effects and 3.5 to define significant QTLs. These thresholds balance mapping accuracy with minimizing false-positives and the omission of minor-effect QTLs. Interval mapping identified 18 QTLs associated with fruit titratable acidity in *C. humilis*, of which 10 were major-effect QTLs concentrated in LG2 and LG5. The distribution of QTLs showed similarities to and distinctions from fruit acidity-related QTLs in other fruit trees. For example, QTLs controlling acidity traits in peach have been mapped to LG5 [45], which concurs partly with the findings of this research. This suggests that the genetic regulation of fruit acidity may involve partially conserved genetic mechanisms across different fruit tree species. Due to differences in genetic background, genome structure, and evolutionary history in different fruit tree species, QTL mapping results for fruit acidity also demonstrate obvious variation [46]. For example, in studies on apricot [47], pear [28], and apple [40], the distribution and number of QTLs associated with fruit titratable acidity vary. Meanwhile, fruit acidity is not an independent trait, as it may be influenced by soluble solids and morphological traits [48]. The research results of Guo et al. indicated that the titratable acid content of the *C. humilis* was significantly correlated with soluble solids (*p* < 0.05), but the correlation coefficients were relatively low, and there was no correlation with other trait indicators. It is speculated that the titratable acid content of the *C. humilis* is less affected by other related fruit traits and is relatively genetically independent, with fruit acidity primarily determined by relatively independent genes [34]. The QTLs for *C. humilis* fruit acidity identified in the current study showed inconsistent intervals between 2021 and 2022, potentially due to environmental sensitivity to quantitative traits: differences in climatic conditions, pest and disease incidence, and harvest periods between 2021 and 2022 may have altered fruit acidity, affecting QTL mapping results. Yao [49] also found that quantitative traits were highly complex and environmentally influenced. Therefore, stable QTLs represent a similarity across years; they still provide valuable insight into genetic regulatory mechanisms underlying *C. humilis* fruit acidity. The inconsistency in QTL mapping results for fruit acidity in *C. humilis* observed between the two years of this study reveals the environmental sensitivity of the genetic regulation underlying this trait. The environment-sensitive QTLs newly identified across the years provide novel insights into unraveling the mechanisms of environmental adaptation governing fruit acidity. Future research should conduct multi-year and multi-location experiments, integrate association analyses between environmental factors and QTL mapping results, and construct a “genotype-environment-phenotype” regulatory network. This approach will enhance the accuracy and stability of QTL mapping, thereby offering more comprehensive theoretical support for the improvement of fruit quality in *C. humilis*.

Candidate gene screening in this study was strictly confined to the mapped acidity-related QTL intervals, and focused primarily on 11 major QTLs on LG2 and LG5 (LOD ≥ 3.5, PVE 8.6–31.13%) for analysis. By aligning the genetic intervals of these QTLs with the physical coordinates of the *C. humilis* reference genome, two candidate genes associated with organic acid metabolism were identified within the major-effect QTL interval of 21TA-5 (89.056–90.97 cm) on LG2: a phosphoester phosphatase gene (*MSTRG.15867*) and a MATE transmembrane transporter (*MSTRG.17104*). The relative phosphoester phosphatase gene expression level aligned with the variation in titratable acid content and was strongly correlated (0.93; *p* < 0.01). This suggests that this gene is crucial for regulating fruit acidity in *C. humilis*. It is purported to influence fruit acidity by participating in organic acid metabolic pathways, such as synthesis regulation, decomposition, or transport of organic acids. Phosphatases may be involved in phosphorus metabolism-related signal transduction, influencing organic acid accumulation or degradation [50]. However, the specific regulatory mechanism remains unclear and requires further comprehensive verification using techniques such as gene editing and transgenesis.

MATE transmembrane transporters may regulate fruit acidity by mediating organic acid intracellular transport and influencing their distribution among different organelles or cells [51]. The gene identified in the current study was located within the QTL interval. Although the correlation between its expression level and fruit titratable acid content has not been verified, its potential role in regulating *C. humilis* fruit acidity remains plausible. Further studies, including gene expression analysis and protein function verification, are needed to elucidate its specific regulatory mechanism in *C. humilis* fruit acidity.

However, despite its contribution, this study had several limitations. Despite the high marker density of the genetic map, some linkage groups (e.g., LG4) had relatively large gaps, which may lead to the omission of some QTLs. Candidate gene functions were only inferred based on expression correlations without in vivo and in vitro validation. Non-coding RNAs or regulatory elements within QTL intervals were not analyzed, potentially ignoring epigenetic regulatory mechanisms.

Future research can be improved in three respects: applying higher-density sequencing and fine mapping to close gaps between and refine candidate gene regions; verifying candidate gene functions using gene editing or transgenic technologies to clarify their specific roles in acidity regulation; and integrating metabolome data to analyze the associations between candidate genes and organic acid metabolites (e.g., citric and malic acid) to elucidate related metabolic pathways.

## 5. Conclusions

A highly saturated and precise genetic linkage map was constructed for *C. humilis*, forming a strong basis for genomic research in the plant. Using this map and two years of phenotypic data, 18 QTLs associated with fruit titratable acidity were identified, including 11 major-effect loci concentrated on LG2 and LG5. These findings elucidate the genetic mechanisms underlying fruit acidity. A phosphoester phosphatase gene and a MATE transmembrane transporter gene were selected as candidate genes, with the former strongly correlated to acidity variation. The study provides theoretical and technical support for marker-assisted breeding and accelerates the development of high-quality, low-acid *C. humilis* cultivars.

## Figures and Tables

**Table 1:** Summary of the genetic map characteristics for Nongda 4 and DS-1.

Linkage Group	SNP Number	Total Distance (cm)	Average Distance (cm)	Max Gap (cm)	Gaps < 5 cm (%)
LG1	465	79.45	0.17	3.35	100
LG2	389	142.71	0.37	18.23	98.97
LG3	384	77.04	0.2	2.87	100
LG4	333	92.18	0.28	24.22	99.7
LG5	305	79.2	0.26	2.39	100
LG6	265	90.52	0.34	20.17	99.62
LG7	197	57.01	0.29	12.71	98.98
LG8	153	54.6	0.36	5.28	98.68
Total	2491	672.71	-	-	-
Average	311.38	84.09	0.27	11.15	99.49

SNP, single-nucleotide polymorphism.

**Table 2:** Quantitative trait locus (QTL) mapping results of fruit acidity based on the genetic map.

Year	Linkage Group	QTL	Confidence Interval (cm)	Mark Number	LOD	PVE (%)
2021	LG2	21TA-1	69.433–69.912	6	3.87	12.03
	LG2	21TA-2	74.218–77.568	16	3.86	12.01
	LG2	21TA-3	84.51–85.467	4	3.33	10.45
	LG2	21TA-4	86.663–87.381	6	3.25	10.19
	LG2	21TA-5	89.056–90.97	13	3.41	10.69
	LG2	21TA-6	91.209–92.405	10	3.68	11.45
	LG2	21TA-7	94.08–94.319	3	3.91	12.13
	LG5	21TA-8	42.349–42.588	3	3.47	10.83
	LG5	21TA-9	45.7–45.94	3	3.56	11.13
	LG5	21TA-10	48.093–48.811	8	5.43	16.44
	LG5	21TA-11	50.007–59.338	35	6.29	18.71
	LG5	21TA-12	60.056–61.971	10	11.28	31.13
	LG5	21TA-13	63.407–79.197	55	8.36	24.06
2022	LG1	22TA-1	29.432	3	3.86	8.7
	LG2	22TA-2	60.336	1	3.81	8.6
	LG3	22TA-3	48.332	1	3.14	7.1
	LG5	22TA-4	34.693	1	3.23	7.3
	LG6	22TA-5	1.436	1	3.45	7.8

LOD, logarithm (base 10) of odds; PVE, phenotypic variation explained.

**Table 3:** Correlation analysis of titratable acid content and phosphatidate phosphatase gene expression during Chinese dwarf cherry fruit development.

Correlation coefficient	Phosphatidate phosphatase gene (*FPKM*)
Titratable acid content	0.93 **

FPKM, fragments per kilobase of exon per million mapped fragments; ** indicates a significant difference at the ** *p* < 0.01 level.

## Data Availability

Data will be made available upon request to the corresponding author.

## Supplementary Materials

The following supporting information can be downloaded at: https://www.mdpi.com/article/10.3390/genes16101157/s1, Table S1: Sequencing data of parents and F1 progenies; Table S2: Sequencing data and reference genome comparison.

## Abbreviations

The following abbreviations are used in this manuscript:

RAD-seq	restriction site-associated DNA sequencing
SNP	single-nucleotide polymorphism
QTL	quantitative trait locus
LOD	logarithm of the odds
PVE	phenotypic variation explained

## References

[1] Mo C., Li W.D., He Y.X., Ye L.Q., Zhang Z.S., Jin J.S. (2015). Variability in the sugar and organic acid composition of the fruit of 57 genotypes of Chinese Dwarf Cherry [*Cerasus humilis* (Bge.) Sok]. J. Hortic. Sci. Biotechnol..

[2] Ren J., Sun L.N., Zhang Q.Y., Song X.S. (2016). Drought tolerance is correlated with the activity of antioxidant enzymes in *Cerasus humilis* seedlings. BioMed Res. Int..

[3] Yang R., Yang Y., Hu Y., Yin L., Qu P., Wang P., Mu X., Zhang S., Xie P., Cheng C. (2023). Comparison of bioactive compounds and antioxidant activities in differentially pigmented *Cerasus humilis* fruits. Molecules.

[4] Ye L.Q., Yang C.X., Li W.D., Hao J.B., Sun M., Zhang J.R., Zhang Z.S. (2017). Evaluation of volatile compounds from Chinese Dwarf Cherry (*Cerasus humilis* (Bge.) Sok.) germplasms by headspace solid-phase micro-extraction and gas chromatography-mass spectrometry. Food Chem..

[5] Mu X.P., Aryal N., Du J.M., Du J.J. (2015). Oil content and fatty acid composition of the kernels of 31 different cultivars of Chinese dwarf cherry [*Cerasus humilis* (Bge.) Sok]. J. Hortic. Sci. Biotechnol..

[6] Li W.D., Li O., Mo C., Jiang Y.S., He Y., Zhang A.R., Chen L.M., Jin J.S. (2014). Mineral element composition of 27 Chinese dwarf cherry [*Cerasus humilis* (Bge.) Sok.] genotypes collected in China. J. Hortic. Sci. Biotechnol..

[7] Ji X.L., Zhang M.Y., Wang D., Li Z., Lang S.Y., Song X.S. (2023). Genome-wide identification of WD40 superfamily in *Cerasus humilis* and functional characteristics of ChTTG1. Int. J. Biol. Macromol..

[8] Guo C.Z., Wang P.F., Zhang J.C., Guo X., Mu X.P., Du J.J. (2022). Organic acid metabolism in Chinese dwarf cherry [*Cerasus humilis* (Bge.) Sok.] is controlled by a complex gene regulatory network. Front. Plant Sci..

[9] Mohd Shaha F.R., Liew P.L., Zaman F.Q., Nulit R., Barin J., Rolland J., Yong H.Y., Boon S.H. (2024). Genotyping by sequencing for the construction of oil palm (*Elaeis guineensis* Jacq.) genetic linkage map and mapping of yield related quantitative trait loci. PeerJ.

[10] Wang W., Xu Z., Qian L., Hang S., Niu Y., Shen C., Wei Y., Liu B. (2024). Genetic mapping and validation of QTL controlling fruit diameter in cucumber. BMC Plant Biol..

[11] Kumar R., Das S.P., Choudhury B.U., Kumar A., Prakash N.R., Verma R., Chakraborti M., Devi A.G., Bhattacharjee B., Das R. (2024). Advances in genomic tools for plant breeding: Harnessing DNA molecular markers, genomic selection, and genome editing. Biol. Res..

[12] Davey J.W., Hohenlohe P.A., Etter P.D., Boone J.Q., Catchen J.M., Blaxter M.L. (2011). Genome-wide genetic marker discovery and genotyping using next-generation sequencing. Nat. Rev. Genet..

[13] Antanaviciute L., Fernández-Fernández F., Jansen J., Banchi E., Evans K.M., Viola R., Velasco R., Dunwell J.M., Troggio M., Sargent D.J. (2012). Development of a dense SNP-based linkage map of an apple rootstock progeny using the *Malus Infinium* whole genome genotyping array. BMC Genom..

[14] Su K., Guo Y.S., Zhong W.H., Lin H., Liu Z.D., Li K., Li Y.Y., Guo X.W. (2021). High-density genetic linkage map construction and white rot resistance quantitative trait loci mapping for genus *Vitis* based on restriction site-associated DNA sequencing. Phytopathology.

[15] Xie Y.H., Feng Y., Chen Q., Zhao F.K., Zhou S.J., Ding Y., Song X.L., Li P., Wang B.H. (2019). Genome-wide association analysis of salt tolerance QTLs with SNP markers in maize (*Zea mays* L.). Genes Genom..

[16] Pereira L., Ruggieri V., Pérez S., Alexiou K.G., Fernández M., Jahrmann T., Pujol M., Garcia-Mas J. (2018). QTL mapping of melon fruit quality traits using a high-density GBS-based genetic map. BMC Plant Biol..

[17] Barchi L., Lanteri S., Portis E., Valè G., Volante A., Pulcini L., Ciriaci T., Acciarri N., Barbierato V., Toppino L. (2012). A RAD tag-derived marker based eggplant linkage map and the location of QTLs determining anthocyanin pigmentation. PLoS ONE.

[18] Jia J.Z., Zhao S.C., Kong X.Y., Li Y.R., Zhao G.Y., He W.M., Appels R., Pfeifer M., Tao Y., Zhang X. (2013). *Aegilops tauschii* draft genome sequence reveals a gene repertoire for wheat adaptation. Nature.

[19] Díaz-Arce N., Rodríguez-Ezpeleta N. (2019). Selecting RAD-seq data analysis parameters for population genetics: The more the better?. Front. Genet..

[20] García-Gómez B., Salazar J.A., Nicolás-Almansa M., Razi M., Rubio M., Ruiz D., Martínez-Gómez P. (2021). Molecular Bases of Fruit Quality in Prunus Species: An Integrated Genomic, Transcriptomic, and Metabolic Review with a Breeding Perspective. Int. J. Mol. Sci..

[21] Argyris J.M., Díaz A., Ruggieri V., Fernández M., Jahrmann T., Gibon Y., Picó B., Martín-Hernández A.M., Monforte A.J., Garcia-Mas J. (2017). QTL analyses in multiple populations employed for the fine mapping and identification of candidate genes at a locus affecting sugar accumulation in melon (*Cucumis melo* L.). Front. Plant Sci..

[22] Zeng Y.L., Wang M.Y., Hunter D.C., Matich A.J., McAtee P.A., Knäbel M., Hamiaux C., Popowski E.A., Jaeger S.R., Nieuwenhuizen N.J. (2020). Sensory-directed genetic and biochemical characterization of volatile terpene production in kiwifruit. Plant Physiol..

[23] Diaz-Garcia L., Schlautman B., Covarrubias-Pazaran G., Maule A., Johnson-Cicalese J., Grygleski E., Vorsa N., Zalapa J. (2018). Massive phenotyping of multiple cranberry populations reveals novel QTLs for fruit anthocyanin content and other important chemical traits. Mol. Genet. Genom..

[24] Tang H.X., Pei G.Y., Zhang Q., Wang Z.T. (2023). QTL mapping analysis of jujube fruit-related traits. Acta Hortic. Sin..

[25] Jiang F.C., Yang L., Zhang J.H., Zhang M.L., Yu W.J., Sun H.Y. (2025). QTL mapping and screening of major-effect genes regulating organic acid accumulation in apricot fruit. Acta Hortic. Sin..

[26] Yamamoto T., Kimura T., Shoda M., Imai T., Saito T., Sawamura Y., Kotobuki K., Hayashi T., Matsuta N. (2002). Genetic linkage maps constructed by using an interspecific cross between Japanese and European pears. Theor. Appl. Genet..

[27] Gangadhara Rao P., Behera T.K., Gaikwad A.B., Munshi A.D., Srivastava A., Boopalakrishnan G. (2021). Vinod Genetic analysis and QTL mapping of yield and fruit traits in bitter gourd (*Momordica charantia* L.). Sci. Rep..

[28] Qin M.F., Li L.T., Singh J., Sun M.Y., Bai B., Li S.W., Ni J.P., Zhang J.Y., Zhang X., Wei W.L. (2022). Construction of a high-density bin-map and identification of fruit quality-related quantitative trait loci and functional genes in pear. Hortic. Res..

[29] Shi P., Xu Z., Zhang S.Y., Wang X.J., Ma X.F., Zheng J.C., Xing L.B., Zhang D., Ma J.J., Han M.Y. (2020). Construction of a high-density SNP-based genetic map and identification of fruit-related QTLs and candidate genes in peach [*Prunus persica* (L.) Batsch]. BMC Plant Biol..

[30] Battistoni B., Salazar J., Vega W., Valderrama-Soto D., Jiménez-Muñoz P., Sepúlveda-González A., Ahumada S., Cho I., Gardana C.S., Morales H. (2022). An upgraded, highly-saturated linkage map of Japanese plum (*Prunus salicina* Lindl.), and identification of a new major locus controlling the flavan-3-ol composition in fruits. Front. Plant Sci..

[31] Oh S., Ahn S., Han H., Kim K., Kim S.A., Kim D. (2023). Genetic linkage maps and QTLs associated with fruit skin color and acidity in apple (*Malus* × *domestica*). Hortic. Environ. Biotechnol..

[32] Wang P.F., Yi S.K., Mu X.P., Zhang J.C., Du J.J. (2020). Chromosome-level genome assembly of *Cerasus humilis* Using PacBio and Hi-C Technologies. Front. Genet..

[33] Zhu H.H., Zhou X. (2020). Statistical methods for SNP heritability estimation and partition: A review. Comput. Struct. Biotechnol. J..

[34] Guo C.Z. (2023). Genetic Analysis of Fruit Organic Acids, Screening and Functional Verification of Key Organic Acid Metabolism Regulating Genes in Chinese Dwarf Cherry. Ph.D. Dissertation.

[35] Catchen J.M., Amores A., Hohenlohe P., Cresko W., Postlethwait J.H. (2011). Stacks: Building and genotyping loci de novo from short-read sequences. G3 Genes|Genomes|Genet..

[36] Rastas P., Paulin L., Hanski I., Lehtonen R., Auvinen P. (2013). Lep-MAP: Fast and accurate linkage map construction for large SNP datasets. Bioinformatics.

[37] Jiang Y.M., Dong L., Li H.Q., Liu Y.N., Wang X.D., Liu G.Q. (2024). Genetic linkage map construction and QTL analysis for plant height in proso millet (*Panicum miliaceum* L.). Theor. Appl. Genet..

[38] Wang Y., Georgi L.L., Reighard G.L., Scorza R., Abbott A.G. (2002). Genetic mapping of the evergrowing gene in peach [*Prunus persica* (L.) Batsch]. J. Hered..

[39] Wang Y., Georgi L.L., Zhebentyayeva T.N., Reighard G.L., Scorza R., Abbott A.G. (2002). High-throughput targeted SSR marker development in peach (*Prunus persica*). Genome.

[40] Sun R., Chang Y., Yang F., Wang Y., Li H., Zhao Y., Chen D., Wu T., Zhang X., Han Z. (2015). A dense SNP genetic map constructed using restriction site-associated DNA sequencing enables detection of QTLs controlling apple fruit quality. BMC Genom..

[41] Li H., Chen A., Tang H., Luan M. (2024). High-density genetic map construction and QTL analysis of the first flower node in kenaf using RAD-seq. BMC Plant Biol..

[42] Wang D.S., Cheng B.B., Zhang J.J. (2024). High-density genetic map and quantitative trait loci map of skin color in hawthorn (*Crataegus pinnatifida* bge. Var. major N.E.Br.). Front. Genet..

[43] Salazar J.A., Pacheco I., Zapata P., Shinya P., Ruiz D., Martinez-Gomz P., Infante R. (2020). Identification of loci controlling phenology, fruit quality and post-harvest quantitative parameters in Japanese plum (*Prunus salicina* Lindl.). Postharvest Biol. Technol..

[44] Powder K.E., Clifton N.J. (2020). Quantitative Trait Loci (QTL) mapping. Methods in Molecular Biology.

[45] Rawandoozi Z.J., Hartmann T.P., Carpenedo S., Gasic K., Linge C.d.S., Cai L., Van de Weg E., Byrne D.H. (2020). Identification and characterization of QTLs for fruit quality traits in peach through a multi-family approach. BMC Genom..

[46] Maan S.S., Brar J.S., Mittal A., Gill M.I.S., Arora N.K., Sohi H.S., Chhuneja P., Dhillon G.S., Singh N., Thakur S. (2023). Construction of a genetic linkage map and QTL mapping of fruit quality traits in guava (*Psidium guajava* L.). Front. Plant Sci..

[47] Dondini L., Domenichini C., Dong Y., Gennari F., Bassi D., Foschi S., Lama M., Adami M., De Franceschi P., Cervellati C. (2022). Quantitative trait loci mapping and identification of candidate genes linked to fruit acidity in apricot (*Prunus armeniaca* L.). Front. Plant Sci..

[48] Doroshenko T.N., Chumakov S.S., Satibalov A.V., Dobrenkov E.A. (2008). Physiological aspects of improving fruit quality in apple plantings. Russ. Agric. Sci..

[49] Yao Y.J., You Q.B., Duan G.Z., Ren J.J., Chu S.S., Zhao J.Q., Li X., Zhou X.N., Jiao Y.Q. (2020). Quantitative trait loci analysis of seed oil content and composition of wild and cultivated soybean. BMC Plant Biol..

[50] Wu S., Li M., Zhang C., Tan Q., Yang X., Sun X., Pan Z., Deng X., Hu C. (2021). Effects of phosphorus on fruit soluble sugar and citric acid accumulations in citrus. Plant Physiol. Biochem..

[51] Dong B., Meng D., Song Z., Cao H., Du T., Qi M., Wang S., Xue J., Yang Q., Fu Y. (2024). CcNFYB3-CcMATE35 and LncRNA CcLTCS-CcCS modules jointly regulate the efflux and synthesis of citrate to enhance aluminium tolerance in pigeon pea. Plant Biotechnol. J..

