# Supplementary material for: Quantitative Trait Locus Mapping and Candidate Gene Identification for Fruit Acidity in Chinese Dwarf Cherry (Cerasus humilis) Using a High-Density Genetic Map
Source: Genes (Basel). 2025 Sep 29;16(10):1157. doi: 10.3390/genes16101157 (PMC12563595; doi:10.3390/genes16101157)
Supplement: Supplementary file 1 [file genes-16-01157-s001.zip › genes-3872645-supplementary.pdf]

Supplementary Material

Supplementary Table S1. Sequencing data of parents and F_1 progenies.

Sample	Total Clean Reads	Total Clean Bases	Q30 Percentage (%)	GC Percentage (%)
Nongda 4	5472088	793098936	91.15	41.61
DS-1	12168660	1779485943	90.87	41.7
F_1 Progenies	1642084928	239289096194	90.65	40.82
Total	1659725676	241861681073	90.89	41.38

Note: Total Clean Reads: the number of reads after filtering; Total Clean Bases: the number of bases after filtering; Q30 Percentage: the percentage of bases whose sequencing quality value is ≥30; GC Percentage: the percentage of G and C bases in sequencing results.

Supplementary Table S2. Sequencing data and reference genome comparison.

Sample	Clean Reads	Mapped-rate (%)	Average sequencing depth
Nongda 4	5667060	95.83	3.61
DS-1	12719448	95.03	8.17
F1 Progenies	1642084928	239289096194	5.25
